# Dupilumab-Associated Ocular Surface Disease in a Local Asian Chinese Population

**DOI:** 10.7759/cureus.102635

**Published:** 2026-01-30

**Authors:** Anita L Li, Andrea C Au, Vivian W Ho, David C Luk, Kai Wang Kenneth Li

**Affiliations:** 1 Department of Ophthalmology, United Christian Hospital, Hong Kong, HKG; 2 Department of Pediatrics, United Christian Hospital, Hong Kong, HKG

**Keywords:** adverse effects, asian population, atopic dermatitis, dupilumab, ocular surface disease

## Abstract

Background: This is a retrospective study evaluating the incidence, clinical characteristics, and risk factors of dupilumab-associated ocular surface disease (DAOSD) in a local Asian Chinese population.

Methodology: A retrospective study of patients prescribed dupilumab for AD between January 2020 and May 2025 at a regional hospital. DAOSD was defined as new-onset ocular surface symptoms post-treatment. Statistical analysis included descriptive statistics, binary logistic regression (age, sex, scoring atopic dermatitis (SCORAD), treatment duration/dosage), and Fisher’s exact test for categorical variables (IBM SPSS v22, IBM Corp., Armonk, NY).

Results: Of 43 included patients (mean age: <25 years, 83.7%; mean treatment duration: 418 days), 11 (25.6%) developed DAOSD (mean onset: 1.67 months). Symptoms were mild (itching, 8, 81.8%; redness, 5, 45.5%; dry eye, 4, 36.4%); 10 (90.9%) required only topical lubricants/antihistamines. No patients discontinued dupilumab due to DAOSD. Logistic regression showed no significant associations between DAOSD and age, sex, SCORAD, dosage, or duration (*P* > 0.05). However, Fisher’s test revealed a significant protective association with prior allergic eye disease (0% DAOSD vs. 33.3% in those without; *P *= 0.043).

Conclusions: The incidence of DAOSD in this Asian cohort was slightly lower than that in Caucasian populations, with predominantly mild symptoms. Prior allergic eye disease may confer protection against DAOSD, possibly due to prophylactic ocular therapies. Larger studies are needed to validate these findings.

## Introduction

Atopic dermatitis (AD) is a chronic, pruritic inflammatory skin disease that disproportionately affects people of color [1], who tend to experience greater disease severity and burden than their Caucasian counterparts. The prevalence of AD is notably high in Asian populations, with studies reporting rates of 10%-20% in children and adolescents in East Asia, including China [2,3]. Dupilumab, a monoclonal antibody targeting interleukin-4 and interleukin-13 signaling, has revolutionized the treatment of moderate-to-severe AD. Despite its efficacy, dupilumab-associated ocular surface disease (DAOSD) remains one of the most frequently reported adverse effects. Proposed mechanisms for DAOSD include the inhibition of interleukin-4/-13 pathways critical for conjunctival goblet cell function and mucin production, leading to tear film instability and ocular surface inflammation [4].

While studies in Caucasian populations suggest a DAOSD incidence of approximately 30% [5], data on Asian and other underrepresented groups remain scarce, a critical gap, particularly given the higher prevalence of AD in these populations and potential ethnic variations in treatment response. As dupilumab use increases globally, understanding the incidence, clinical spectrum, and risk factors of DAOSD across different demographic groups is essential for optimizing patient care. This study aims to evaluate the incidence, clinical characteristics, and risk factors of DAOSD in a local Asian Chinese population.

## Materials and methods

We conducted a retrospective analysis of all patients prescribed dupilumab (subcutaneous injection) for AD between January 2020 and May 2025 at a regional hospital. Patient records were identified from the hospital pharmacy database. All age groups were included. Clinical data and demographics were collected from electronic health records.

All patients prescribed dupilumab for AD were included. Patients were defined as having DAOSD if they developed new-onset ocular symptoms (e.g., itching, redness, dry eye, stinging, burning, pain, foreign body sensation, or photophobia) after initiating dupilumab therapy, as documented in clinical notes. This definition aligns with clinical criteria commonly used in prior studies [2,3]. Patients with pre-existing ocular surface disease unrelated to dupilumab were not classified as having DAOSD unless they experienced a significant new onset or worsening of symptoms after treatment initiation.

All statistical analyses were performed using IBM SPSS Statistics (version 22.0, IBM Corp., Armonk, NY). Descriptive statistics were used to summarize demographic and clinical characteristics. The primary outcome was the incidence of DAOSD. Risk factor analysis was performed using binary logistic regression for continuous/polytomous variables (age, SCORAD, treatment duration, dosage) and Fisher’s exact test for categorical variables (e.g., history of allergic eye disease). A two-tailed *P*-value <0.05 was considered statistically significant.

## Results

A total of 45 cases that were prescribed dupilumab between January 2020 and May 2025 were identified. Two cases were excluded due to not being on dupilumab for AD. The demographics are presented in Table 1. Out of 43 subjects, 11 (25.6%) developed ocular surface symptoms while being on dupilumab. Among symptomatic patients, the main symptoms reported were itching (*n* = 8, 81.8%), redness (*n* = 5, 45.5%), and dry eye (*n* = 4, 36.4%). Of the patients with DAOSD, 10 (90.9%) were treated with lubricants and topical antihistamines alone. One patient required treatment with topical steroids and steroid-sparing agents (Cyclosporine A/Tacrolimus). None of the patients in the cohort discontinued dupilumab due to ocular surface symptoms. 

**Table 1:** Demographic and clinical characteristics of patients with atopic dermatitis treated with dupilumab (n = 43).

Characteristics	Value
Total cases	43
Excluded cases	2 (not prescribed for atopic dermatitis)
Demographics	
Sex, n (%)	
Female	22 (51.2)
Male	21 (48.8)
Race: ethnic Chinese	43 (100)
Age distribution, n (%)	
<25 years	36 (83.7)
≥25 years	7 (16.3)
Age range (years)	2-66
Dupilumab treatment	
Mean duration, days (SD)	418 (168)
Dupilumab-associated ocular surface disease (DAOSD)	
Number of cases, n (%)	11 (25.6)
Mean time to onset, months (SD)	1.67 (6.02)
Discontinued due to DAOSD, n (%)	0 (0.0)

Logistic regression showed that eczema score (SCORAD [6]), age, gender, dose, and duration of treatment were not significantly associated with the presence of dupilumab-associated ocular surface disease (Table 2). Due to complete separation (no DAOSD cases among patients with prior allergic eye disease), this variable was excluded from regression. A separate analysis using Fisher’s exact test showed a significant association: none of the patients with a history of allergic eye disease developed DAOSD, compared with 11 of 33 patients (33.3%) without a history of allergic eye disease (*P* = 0.043).

**Table 2:** Logistic regression analysis of factors associated with DAOSD. ^†^Gender coded as 0 = Female, 1 = Male. DAOSD, dupilumab-associated ocular surface disease

Variable	B (SE)	Wald χ²	P-value	Odds ratio (Exp(B))	95% CI for Exp(B)
Age	0.137 (0.141)	0.941	0.332	1.146	0.869-1.512
Gender^†^	1.916 (1.348)	2.021	0.155	6.794	0.481-95.934
SCORAD	0.005 (0.031)	0.027	0.870	1.005	0.946-1.068
Duration	0.009 (0.005)	2.641	0.104	1.009	0.998-1.019
Dosage	-0.008 (0.013)	0.401	0.527	0.992	0.967-1.018

## Discussion

The incidence of DAOSD (*n* = 11, 25.6%) in this Asian population was slightly lower than the approximately 30% reported in several Caucasian population studies [5,7-8]. This aligns with limited data from other Asian cohorts; a study in a Korean population reported an incidence of 28.9% [9]. Notably, AD is highly prevalent in Asian populations [2,3], making the characterization of treatment-related adverse effects in this group particularly relevant.

The symptoms of DAOSD in this cohort were mild, primarily itching, redness, and dry eyes. Most DAOSD patients were managed with lubricant and antihistamine eye drops alone, with only one requiring topical steroid or steroid-sparing agents. Despite a mean treatment duration exceeding one year, no patient discontinued dupilumab due to DAOSD, consistent with findings by Yap et al. [10], in a pediatric Asian cohort. In our review, other ocular complications (e.g., uveitis, keratitis) were not found, suggesting that in this cohort, DAOSD manifested primarily as mild ocular surface inflammation.

Regarding risk factors, Shim et al. found an association with older age (>25 years) [9], which was not observed in our cohort. This discrepancy may be due to our predominantly young population (<25 years: 83.7%) and limited sample size. Our study’s primary limitation is its small sample size from a single institution, which reduces statistical power.

Despite the small sample, a significant finding was that no patient, 0 (0%) of 10, with a history of allergic eye disease developed DAOSD, compared to 11 (33.3%) of 33 patients without such a history. While requiring validation in larger studies, this may suggest a protective effect, possibly because these patients were already using prophylactic lubricant or antihistamine eye drops. This hypothesis is further supported by the study by Yap et al. [10], which reported a relatively low DAOSD incidence (7.4%), with most patients using prophylactic lubricants. Evidence for a potential protective effect, however, remains lacking in the current literature [11].

## Conclusions

In this predominantly young, Asian Chinese cohort with AD, the incidence of DAOSD was slightly lower than in Caucasian populations. DAOSD symptoms were typically mild and did not necessitate discontinuation of dupilumab. A history of allergic eye disease was associated with a significantly lower risk of developing DAOSD, potentially due to concomitant use of topical ocular therapies. These findings contribute to the limited data on DAOSD in Asian populations. Larger, prospective studies are needed to confirm these observations and further elucidate risk factors.
